# Sex differences in the development of vascular and renal lesions in mice with a simultaneous deficiency of *Apoe* and the integrin chain *Itga8*

**DOI:** 10.1186/s13293-017-0141-y

**Published:** 2017-05-30

**Authors:** Ines Marek, Maurizio Canu, Nada Cordasic, Manfred Rauh, Gudrun Volkert, Fabian B. Fahlbusch, Wolfgang Rascher, Karl F. Hilgers, Andrea Hartner, Carlos Menendez-Castro

**Affiliations:** 1 Department of Pediatrics and Adolescent Medicine, University Hospital of Erlangen-Nuernberg, Loschgestrasse 15, 91054 Erlangen, Germany; 2 Department of Nephrology and Hypertension, University Hospital of Erlangen-Nuernberg, Erlangen, Germany

**Keywords:** α8 integrin, *Itga8*, Atherosclerotic lesions, Renal lesions, Knockout mice, Sex differences, Apolipoprotein E, Vascular remodeling

## Abstract

**Background:**

*Apoe*-deficient (*Apoe* ^−/−^) mice develop progressive atherosclerotic lesions with age but no severe renal pathology in the absence of additional challenges. We recently described accelerated atherosclerosis as well as marked renal injury in *Apoe* ^−/−^ mice deficient in the mesenchymal integrin chain *Itga8* (*Itga8* ^−/−^). Here, we used this *Apoe* ^−/−^, *Itga8* ^−/−^ mouse model to investigate the sex differences in the development of atherosclerosis and concomitant renal injury. We hypothesized that aging female mice are protected from vascular and renal damage in this mouse model.

**Methods:**

*Apoe* ^−/−^ mice were backcrossed with *Itga8* ^−/−^ mice. Mice were kept on a normal diet. At the age of 12 months, the aortae and kidneys of male and female *Apoe* ^−/−^ *Itga8* ^+/+^ mice or *Apoe* ^−/−^ *Itga8* ^−/−^ mice were studied. En face preparations of the aorta were stained with Sudan IV (lipid deposition) or von Kossa (calcification). In kidney tissue, immunostaining for collagen IV, CD3, F4/80, and PCNA and real-time PCR analyses for *Il6*, *Vegfa*, *Col1a1* (collagen I), and *Ssp1* (secreted phosphoprotein 1, synonym osteopontin) as well as ER stress markers were performed.

**Results:**

When compared to male mice, *Apoe* ^−/−^ *Itga8* ^+/+^ female mice had a lower body weight, equal serum cholesterol levels, and lower triglyceride levels. However, female mice had increased aortic lipid deposition and more aortic calcifications than males. Male *Apoe* ^−/−^ mice with the additional deficiency of *Itga8* developed increased serum urea, glomerulosclerosis, renal immune cell infiltration, and reduced glomerular cell proliferation. In females of the same genotype, these renal changes were less pronounced and were accompanied by lower expression of interleukin-6 and collagen I, while osteopontin expression was higher and markers of ER stress were not different.

**Conclusions:**

In this model of atherosclerosis, the female sex is a risk factor to develop more severe atherosclerotic lesions, even though serum fat levels are higher in males. In contrast, female mice are protected from renal damage, which is accompanied by attenuated inflammation and matrix deposition. Thus, sex affects vascular and renal injury in a differential manner.

**Electronic supplementary material:**

The online version of this article (doi:10.1186/s13293-017-0141-y) contains supplementary material, which is available to authorized users.

## Background

Mice with a deficiency of *Apoe* develop hypercholesterolemia followed by progressive atherosclerotic lesions [1, 2]. Hypercholesterolemia is a risk factor for renal damage [3, 4], although *Apoe*-deficient mice without an atherogenic diet do not commonly develop overt renal pathologies [5]. We, therefore, crossed *Apoe*-deficient mice with mice which had a deletion of the *Itga8* (alpha8 integrin) gene, which have reduced nephron numbers and are prone to develop renal disease when challenged [6]. Alpha8 integrin is a matrix receptor mainly found on mesenchymal cells like vascular smooth muscle cells and renal mesangial cells [7]. It is known to exert protective effects for the vasculature most likely by inhibiting smooth muscle cell migration in vascular injury [5, 8, 9]. Moreover, the expression of *Itga8* in renal mesangial cells supports their adhesion and thereby confers structural integrity of the glomerular tuft [9]. As a consequence, mice with a deletion of *Itga8* do not develop overt glomerular injury per se, but are prone to develop glomerulosclerosis when challenged. Little is known so far regarding sex-specific differences in integrin signaling; however, some studies suggest that there is an effect of sex [10, 11]. In a previous study, we investigated the vascular and renal phenotype of male mice with a concomitant deficiency of *Apoe* and *Itga8*. These mice developed more severe atherosclerotic lesions than mice only deficient in *Apoe*. Moreover, renal injury became overt in male mice with a concomitant deficiency of *Apoe* and *Itga8* [5].

Sex has an impact on the development and progression of vascular and renal disease [12–14]. A number of experimental studies in *Apoe*-deficient mice detected sex differences in the development and progression of vascular and renal disease [15]. In most cases, the male sex was a risk factor to develop more severe cardiovascular and renal damage [16–19]. On the other hand, studies exist which found females more seriously affected by vascular lesions [20], or alternatively, no significant differences between vascular lesion sizes of males and females were observed [21]. In the present study, we have compared vascular and renal changes in male and female mice with a deletion for both *Apoe* and *Itga8*. We utilized the deficiency of *Itga8* to increase the susceptibility of the *Apoe*-deficient mice to develop glomerulosclerosis. As the progression of glomerulosclerosis is critically influenced by sex hormones [22–24], we hypothesized that female mice are protected from the increased risk for glomerulosclerosis seen in male mice of our experimental atherosclerosis model.

## Methods

### Animal procedures

*Itga8*-deficient mice (*Itga8* ^−/−^, gift from Dr. Ulrich Muller, Scripps Institute, La Jolla, USA) on a mixed genetic background (C57BL/6x129Sv) were crossed with *Apoe*-deficient mice (*Apoe* ^−/−^), which are prone to develop atherosclerotic lesions (Charles River, Sulzfeld, Germany). Animals were kept on a standard rodent maintenance diet (#1320, Altromin, Lage, Germany) containing 4% fat (http://www.altromin.com/fileadmin/downloads/specs/standard/1320.pdf) with free access to tap water in a room maintained at 22 ± 2 °C with a 12-h dark/light cycle. All procedures performed on animals were approved by the local government authorities (Regierung von Mittelfranken, AZ No. 621-2531.31-10/02) and were done in compliance with the DIRECTIVE 2010/63/EU of the European Parliament. Male and female litters of the genotypes *Apoe* ^−/−^ *Itga8* ^+/+^ (*n* = 11 for males and *n* = 12 for females) and *Apoe* ^−/−^ *Itga8* ^−/−^ (*n* = 12 for males and *n* = 18 for females) were used to perform histological analyses at the age of 1 year. For gene expression analyses, additional animals were used to prepare RNA from aortae and kidneys (*Apoe* ^−/−^ *Itga8* ^+/+^: *n* = 6 for males, *n* = 5 for females and *Apoe* ^−/−^ *Itga8* ^−/−^: *n* = 7 for males and *n* = 7 for females). Additional control groups for the evaluation of the effects of an *Itga8* deficiency only were *Apoe* ^+/+^ *Itga8* ^+/+^ (*n* = 11 for males and *n* = 12 for females) and *Apoe* ^+/+^ *Itga8* ^−/−^ at the age of 1 year (*n* = 14 for males and *n* = 11 for females). The time point of sacrifice was chosen based on pilot studies and published data describing the time course of lesion development in this mouse model under standard rodent chow [25]. We also sacrificed some animals at the age of 7 and of 9 months (*n* = 6 per group) to follow the progress of lesion development. Mice were sacrificed between 8:00 in the morning and noon without prior fasting. Blood samples were obtained at sacrifice after isoflurane anesthesia. For vascular analyses, adventitial tissue was removed from the aorta and the distal part of the brachiocephalic trunk. The brachiocephalic trunk was used for preparing paraffin sections. The aorta was prepared en face as described [26]. For kidney analyses, tissue samples were prepared for histology and immunohistochemistry as described [27, 28].

### Serum analyses

Total plasma cholesterol, high-density lipoprotein (HDL), cholesterol, triglycerides, creatinine, and urea were measured using an automatic analyzer, Integra 800 (Roche Diagnostics, Mannheim, Germany). Serum values of low-density lipoprotein (LDL) were calculated using the Friedewald formula [29]. The concentration of testosterone was measured in mouse serum by using liquid chromatography-tandem mass spectrometry (LC–MS/MS) as described in [30]. The steroid testosterone was measured by a modified online SPE-HPLC-MS/MS combined with atmospheric pressure chemical ionization in the positive ion mode.

### En face preparations of the aorta

The aorta was fixed with 4% paraformaldehyde and opened longitudinally after spanning the aortic arch to the iliac bifurcation as described before [26]. Sudan IV staining was performed to detect lipid deposition of atherosclerotic plaques. Von Kossa staining was used to detect calcifications. Native and Sudan IV-stained as well as native and von Kossa-stained aortic preparations were photographed, and the image analysis software MetaVue (Molecular Devices, Sunnyvale, CA, USA) was used to quantify atherosclerotic lesions as a percentage of total aortic surface area. Lipid deposition and calcification were quantified in the aortic arch and the descending aorta separately.

### Vascular histology

The distal part of the brachiocephalic trunk was fixed in 4% paraformaldehyde and embedded in paraffin. Two-micrometer cross sections of the brachiocephalic trunk were stained with PAS and von Kossa to confirm calcifications.

### Immunohistochemistry

For immunohistochemical staining, kidneys were fixed in methyl Carnoy’s solution and embedded in paraffin. Two-micrometer sections were stained as described below. Sections were blocked with 3% H_2_O_2_. Primary antibodies were incubated overnight at the following dilutions: proliferating cell nuclear antigen (PCNA) for proliferating cells (M0879; DAKO) 1:50, F4/80 for macrophages (LMU8949; Linaris, Dossenheim, Germany) 1:50, CD3 for T cells (I7A2; BioLegend, Fell, Germany) 1:300, collagen IV (Southern Biotechnology, Birmingham, AL, USA) 1:500, osteopontin (OPN; sc-21742, Santa Cruz Biotechnology, Heidelberg, Germany) 1:50. For staining with PCNA and osteopontin, a mouse-on-mouse kit was used (M.O.M. Kit, Vector, Burlingame, CA, USA). Appropriate secondary antibodies (Vector) were diluted 1:500, before avidin D peroxidase (Vector) was applied at a dilution of 1:2000. Finally, DAB (Vector) was added, and sections were counterstained with hematoxylin and covered with entellan.

To analyze differences in glomerular proliferation, stained and unstained nuclei were counted and proliferation was expressed as a percentage of positive nuclei/glomerulus. CD3-positive cells (T cells) and F4/80-positive cells (macrophages) were counted in five non-overlapping medium-power cortical views and presented as positive cells/cortical view. Glomerular collagen IV expression was quantified in 20 glomeruli/renal section, as a ratio of stained area/total area of glomerular cross sections, using MetaVue software.

### Isolation of mRNA and real-time PCR

For expression analyses, kidneys and aortae were taken out and immediately snap frozen on liquid nitrogen and stored at −80 °C. Aortic or kidney tissue (1–10 mg) was homogenized in 300–500 μl RLT buffer reagent (Qiagen, Hilden, Germany) with an Ultraturrax for 30 s, and total RNA was extracted with RNeasy® Mini columns (Qiagen) or RNeasy Fibrous Mini Kit (Qiagen) according to the manufacturer’s instructions including RNase-free DNase I treatment on the columns of the RNeasy Mini Kit (Qiagen). RNA concentration was quantified using a NanoDrop ND-1000 spectrophotometer (Thermo Fisher Scientific, Waltham, MA, USA) by measuring the absorbance at 260 nm. Purity of RNA was checked via the A260/A280 nm ratio. TaqMan reverse transcription reagents (Applied Biosystems, Weiterstadt, Germany) with random hexamers as primers were used to obtain first-strand complementary DNA (cDNA). Final concentrations of the reverse transcription master mix were 1× TaqMan RT buffer, 5.5 mM MgCl, 500 μM dNTP mix, 2.5 μM random hexamers, 0.4 U/μl RNAse inhibitor, and 1.2 U/μl MultiScribe RT. RNAse-free water (Qiagen) was adjusted to a final volume of 30 μl per sample. Final RNA concentration in the reaction mixture was adjusted to 3 ng/μl. To test for genomic DNA contamination, reactions without MultiScribe reverse transcriptase were performed as negative controls. Reverse transcription was performed on a thermocycler (TRIO thermoblock, Biometra, Göttingen, Germany) with 10 min at 25 °C, 45 min at 48 °C, 5 min at 95 °C, and hold at 4 °C. cDNA was stored at −20 °C. Reverse transcription products were diluted 1∶1 with dH_2_O. Then, real-time PCR was accomplished with a StepOnePlus Real-Time Cycler (Thermo Fisher Scientific) and Fast SYBR Green (Thermo Fisher Scientific) according to the manufacturer’s protocol in MicroAmp Fast 96-well reaction plates (Thermo Fisher Scientific). Final concentration of the Fast SYBR Green Master Mix was 1× Fast SYBR Green Master Mix buffer, 200 nM forward primer, 200 nM reverse primer, 2.6 μl DNA-free water, and 2 μl cDNA per 10 μl reaction sample/well. The temperature profile included a holding stage of 20 s at 95 °C, then 3 s at 95 °C followed by 30 s at 60 °C. The cycle was repeated 40 times. Melting curves were evaluated to check for primer specificity for the PCR product. NTCs were performed. Real-time PCR data were analyzed using the comparative cycle threshold method with normalizing *C*_t_ to *Rn18s* (18S rRNA). Comparison of *Rn18s* (18S rRNA), *Actb* (beta actin), and *Gapdh* (glyceraldehyde 3-phosphate dehydrogenase) as reference genes showed similar results. See Additional file 1 for the primers used for amplification. All samples were run in triplicates of the same reverse transcription replicate. Primer pairs were designed using the Primer Express software (PerkinElmer, Foster City, CA, USA) except for *Ccl2* (CC-chemokine ligand 2, synonym MCP1) [31] and *Eif2ak3* (eukaryotic translation initiation factor 2-alpha kinase 3, synonym PERK) (designed with PubMed primer software).

### Analysis of data

Data are expressed as mean ± standard error of the mean (SEM), median, and quartile ranges (1.Q/3.Q, IQR). After testing for normality distribution using Shapiro-Wilk’s test, we performed either one-way analysis of variance (ANOVA), followed by the Bonferroni post hoc test, or non-parametric Kruskall-Wallis, followed by Dunn’s test, where appropriate, to assess the differences between the groups using GraphPad Prism software (Version 7, GraphPad Software Inc, San Diego, CA, USA). For select parameters, two-way ANOVA was performed to assess effects of the factors “sex” and “genotype” as well as their interaction (IBM SPSS Statistics 21, Ehningen, Germany). Results were considered significant at *p* < 0.05.

## Results

*Apoe*-deficient (*Apoe* ^−/−^) male and female mice, which are prone to develop atherosclerotic lesions with age, were compared for the extent of vascular plaque formation. *Apoe*-deficient male and female mice with an additional deficiency of *Itga8* (*Apoe* ^−/−^ *Itga8* ^−/−^) were compared for the extent of renal injury. Assessment of body weights revealed significantly lower values in female mice of both genotypes (*Apoe* ^−/−^ *Itga8* ^+/+^ and *Apoe* ^−/−^ *Itga8* ^−/−^) compared to respective males at 1 year of age (Table 1). Relative kidney weights were lower in female mice compared to male mice of the same genotype. A lack in *Itga8* (*Itga8* ^−/−^) resulted in reduced kidney weights in males and females (Table 1). As an indirect marker of chronic arterial hypertension, we also assessed relative left ventricular weights, which were not significantly different between all groups (Table 1). Aortic messenger RNA (mRNA) expression of *Itga8* was quantified to confirm *Itga8* deficiency in double-knockout (*Apoe* ^−/−^ *Itga8* ^−/−^) mice (Table 2). Compared with mice, which were wild type for *Apoe* (*Apoe* ^+/+^ *Itga8* ^+/+^ and *Apoe* ^+/+^ *Itga8* ^−/−^) (Additional file 2), total serum cholesterol as well as LDL cholesterol levels were elevated in *Apoe*-deficient (*Apoe* ^−/−^ *Itga8* ^+/+^ and *Apoe* ^−/−^ *Itga8* ^−/−^) mice (Table 2). The *Itga8* genotype had no influence on serum fat levels within the same *Apoe* genotype (Table 2 and Additional file 2), and mice with a deficiency of only *Itga8* (*Itga8* ^−/−^) did not develop hyperlipidemia (Additional file 2). Serum testosterone levels, which were significantly higher in male mice compared to female mice, did not differ significantly in male mice of the different genotypes (Table 2).

**Table 1:** Anatomical data

	Genotype	Sex	Mean	SEM	Median	Q1	Q3	IQR
Body weight (g)	*Apoe* ^−/−^ *Itga8* ^+/+^	Male	34.4	0.8	34.1	31.7	36.0	4.3
		Female	27.7*	0.9	28.3	25.5	29.0	3.5
	*Apoe* ^−/−^ *Itga8* ^−/−^	Male	31.7	0.6	31.9	30.0	34.1	4.1
		Female	28.4*	0.7	27.9	25.7	31.3	5.6
Absolute kidney weight (g)	*Apoe* ^−/−^ *Itga8* ^+/+^	Male	0.42	0.01	0.44	0.38	0.47	0.09
		Female	0.28*	0.01	0.27	0.26	0.30	0.04
	*Apoe* ^−/−^ *Itga8* ^−/−^	Male	0.29^#^	0.01	0.27	0.26	0.33	0.07
		Female	0.25	0.01	0.26	0.23	0.28	0.05
Relative kidney weight (%)	*Apoe* ^−/−^ *Itga8* ^+/+^	Male	1.23	0.03	1.22	1.12	1.35	0.23
		Female	1.03*	0.03	1.0	0.96	1.08	0.12
	*Apoe* ^−/−^ *Itga8* ^−/−^	Male	0.8^#^	0.03	0.82	0.66	0.93	0.27
		Female	0.76^#^	0.03	0.74	0.63	0.87	0.24
Relative left ventricular weight (%)	*Apoe* ^−/−^ *Itga8* ^+/+^	Male	0.37	0.01	0.37	0.35	0.39	0.04
		Female	0.33	0.01	0.32	0.31	0.35	0.04
	*Apoe* ^−/−^ *Itga8* ^−/−^	Male	0.39	0.01	0.39	0.37	0.41	0.04
		Female	0.36	0.01	0.35	0.32	0.38	0.06

#*p* < 0.05 vs *Itga8* ^+/+^ of the same sex

*p < 0.05 vs male of the same genotype

**Table 2:** Cardiovascular and metabolic parameters

	Genotype	Sex	Mean	SEM	Median	Q1	Q3	IQR
Aortic *Itga8* mRNA expression (fold change)	*Apoe* ^−/−^ *Itga8* ^+/+^	Male	0.96	0.16	0.93	0.59	1.39	0.80
		Female	1.00	0.26	1.05	0.35	1.62	1.27
	*Apoe* ^−/−^ *Itga8* ^−/−^	Male	0.0002^#^	0.00	0.00	0.00	0.00	0.00
		Female	0.05^#^	0.05	0.00	0.00	0.00	0.00
Plasma triglycerides (mg/dl)	*Apoe* ^−/−^ *Itga8* ^+/+^	Male	117	16	102	71.8	154	82.2
		Female	78	7	77.8	56.7	102	45.3
	*Apoe* ^−/−^ *Itga8* ^−/−^	Male	146	24	102	66.9	192	125.1
		Female	111	25	81.3	58.5	114	55.5
Plasma total cholesterol (mg/dl)	*Apoe* ^−/−^ *Itga8* ^+/+^	Male	372	40	453	192	522	330
		Female	406	22	425	345	476	131
	*Apoe* ^−/−^ *Itga8* ^−/−^	Male	393	39	402	235	521	286
		Female	263	28	270	155	363	208
Plasma HDL cholesterol [mg/dl]	*Apoe* ^−/−^ *Itga8* ^+/+^	Male	87	9	87	54	106	52
		Female	96	6	96	83	106	23
	*Apoe* ^−/−^ *Itga8* ^−/−^	Male	100	13	74	63	111	48
		Female	95	7	87	74	101	27
Plasma LDL cholesterol (mg/dl)	*Apoe* ^−/−^ *Itga8* ^+/+^	Male	262	33	320	121	379	258
		Female	295	18	311	251	340	89
	*Apoe* ^−/−^ *Itga8* ^−/−^	Male	263	30	296	142	359	217
		Female	163^#^*	23	159	67	241	174
Plasma testosterone (ng/dl)	*Apoe* ^−/−^ *Itga8* ^+/+^	Male	4	2	0.63	0.32	7.48	7.16
		Female	0.2	0.0	0.15	0.03	0.24	9.21
	*Apoe* ^−/−^ *Itga8* ^−/−^	Male	11	4	12.3	0.77	18.2	17.4
		Female	0.2*	0.1	0.15	0.09	28.1	28.0
Plasma creatinine (μg/dl)	*Apoe* ^−/−^ *Itga8* ^+/+^	Male	52	9	50	20	80	60
		Female	95	9	100	50	120	70
	*Apoe* ^−/−^ *Itga8* ^−/−^	Male	97	8	100	70	130	60
		Female	140	3	110	80	130	50
Plaques of aortic arch (%)	*Apoe* ^−/−^ *Itga8* ^+/+^	Male	17.7	2.34	17.1	11.7	20.6	8.9
		Female	25.7	2.73	25.6	18.7	31.4	12.7
	*Apoe* ^−/−^ *Itga8* ^−/−^	Male	26.6	2.27	25.8	20.3	34.3	14
		Female	21.9	1.76	23.8	15.0	27.7	12.7
Calcification aorta (%)	*Apoe* ^−/−^ *Itga8* ^+/+^	Male	0.04	0.04	0.0	0.0	0.0	0
		Female	2.84*	0.51	2.66	1.58	4.39	2,81
	*Apoe* ^−/−^ *Itga8* ^−/−^	Male	0.85	0.37	0.0	0.0	1.98	1.98
		Female	2.76	0.63	2.56	0.0	3.79	3.79

#*p* < 0.05 vs *Itga8* ^+/+^of the same sex

*p < 0.05 vs male of the same genotype

### Female *Apoe*-deficient (*Apoe*^−/−^*Itga8*^+/+^ and *Apoe*^−/−^*Itga8*^−/−^) mice develop more severe vascular lesions than males, despite similar serum fat levels

**Fig. 1:**
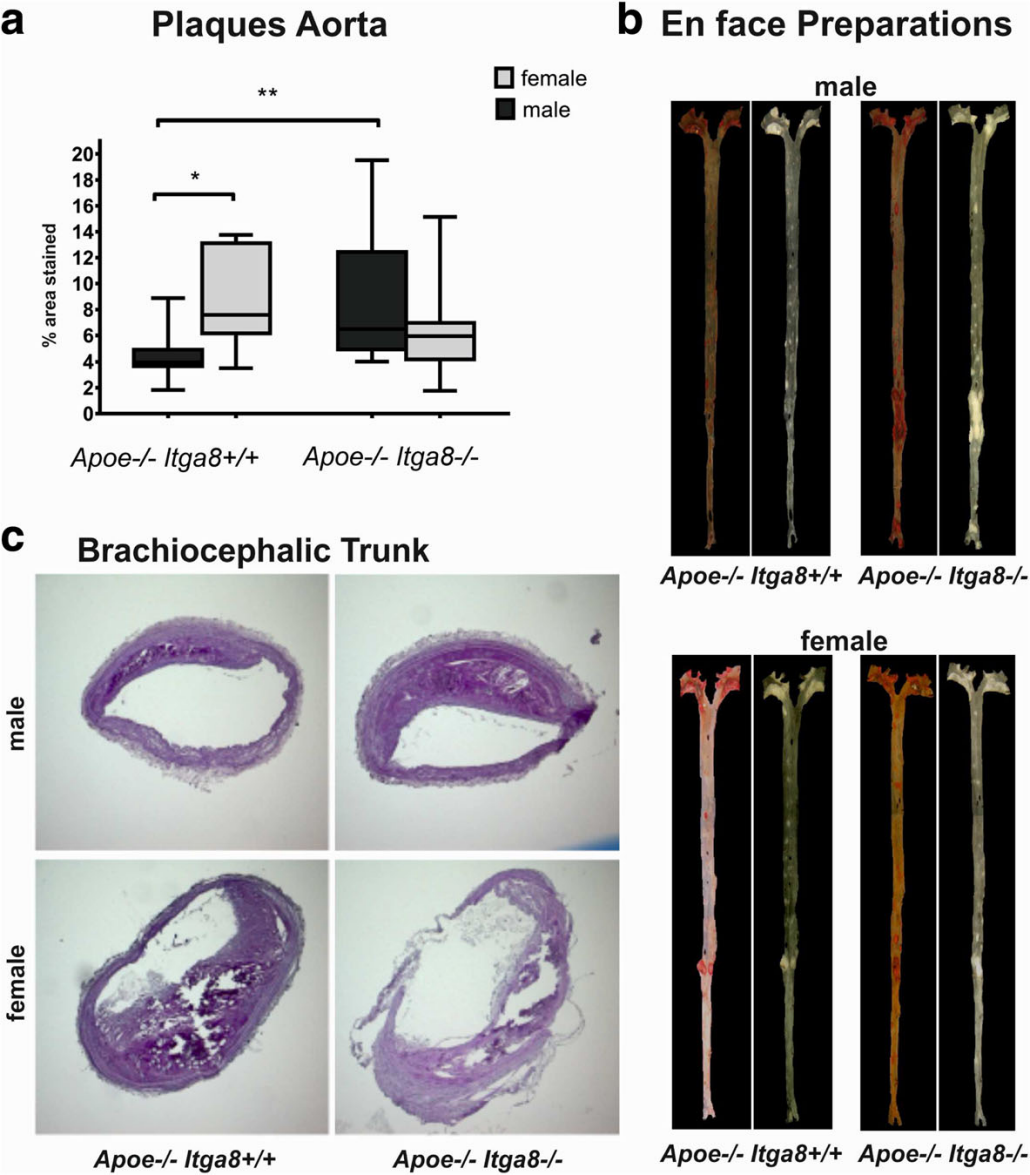
Atherosclerotic plaque formation. **a** Evaluation of aortic plaque formation in male and female *Apoe* ^−/−^ *Itga8* ^+/+^ or *Apoe* ^−/−^ *Itga8* ^−/−^ mice. **b** Exemplary en face preparations of aortae of male and female *Apoe* ^−/−^ *Itga8* ^+/+^ or *Apoe* ^−/−^ *Itga8* ^−/−^ mice, stained with Sudan IV (*left*) or unstained (*right*). **c** Exemplary PAS-stained cross sections of the brachiocephalic trunk of male and female *Apoe* ^−/−^ *Itga8* ^+/+^ or *Apoe* ^−/−^ *Itga8* ^−/−^ mice. *p < 0.05; **p < 0.01

**Fig. 2:**
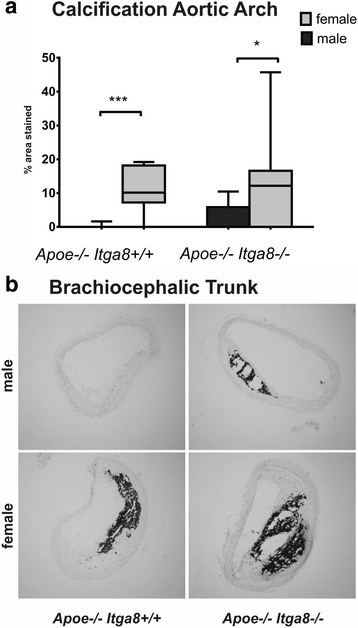
Vascular calcification. **a** Evaluation of en face preparations of aortae of male and female *Apoe* ^−/−^ *Itga8* ^+/+^ or *Apoe* ^−/−^ *Itga8* ^−/−^ mice stained with von Kossa. **b** Exemplary von Kossa-stained cross sections of the brachiocephalic trunk of male and female *Apoe* ^−/−^ *Itga8* ^+/+^ or *Apoe* ^−/−^ *Itga8* ^−/−^ mice. *p < 0.05, ***p < 0.001

Total serum cholesterol levels, serum HDL levels, and serum triglycerides were not significantly different between males and females of the genotypes *Apoe* ^−/−^ *Itga8* ^+/+^ and *Apoe* ^−/−^ *Itga8* ^−/−^ (Table 2). Serum LDL levels were somewhat lower in female mice of the *Apoe* ^−/−^ *Itga8* ^−/−^ genotype. To study the manifestation of atherosclerosis, we assessed the formation of atherosclerotic plaques in the aorta and in the aortic arch. In the aorta, there were significantly more lesions detected in female *Apoe* ^−/−^ *Itga8* ^+/+^ mice, compared to males of the same genotype. In contrast, plaque formation was not different in female and male double-knockout (*Apoe* ^−/−^ *Itga8* ^−/−^) mice, as male *Apoe* ^−/−^ *Itga8* ^−/−^ mice showed an increase in atherosclerotic plaque formation in comparison to male *Apoe* ^−/−^ *Itga8* ^+/+^ (Fig. 1). By two-way ANOVA, there was a significant interaction of sex with genotype regarding aortic plaque formation (see Additional file 3). Mice with a deficiency just of *Itga8* (*Apoe* ^+/+^ *Itga8* ^−/−^) did not develop any atherosclerotic plaques (Additional file 4). The calcification of plaques was much more prominent in female *Apoe*-deficient mice than in males, irrespective of the genotype (Fig. 2 and Additional file 3). In atherosclerotic plaques of female mice, osteopontin staining was much more abundant than in plaques of male mice (Additional file 5) and was predominantly detected at sites of calcification. To track the progress of atherosclerosis, we investigated the aortae of mice at an age of 7 and of 9 months, also. At the age of 7 months, lipid deposition was only weak and did not significantly differ between groups (Additional file 6), while at the age of 9 months, female *Apoe* ^−/−^ *Itga8* ^+/+^ mice displayed an increased atherosclerotic plaque area compared to males of the same genotype (Additional file 6).

### Female *Apoe*-deficient (*Apoe*^−/−^*Itga8*^−/−^) mice are protected from the development of renal injury

**Fig. 3:**
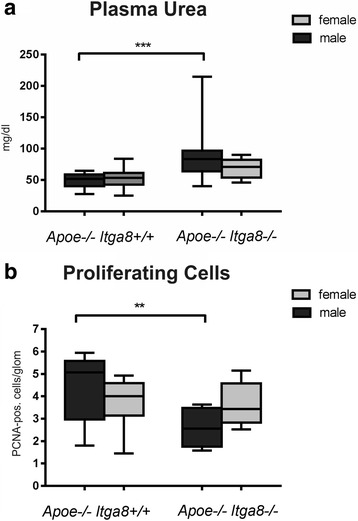
Renal injury. **a** Plasma urea concentration of male and female *Apoe* ^−/−^ *Itga8* ^+/+^ or *Apoe* ^−/−^ *Itga8* ^−/−^ mice. **b** Glomerular cell proliferation in the kidneys of male and female *Apoe* ^−/−^ *Itga8* ^+/+^ or *Apoe* ^−/−^ *Itga8* ^−/−^ mice. Data are presented as means ± SEM. **p < 0.01; ***p < 0.001

**Fig. 4:**
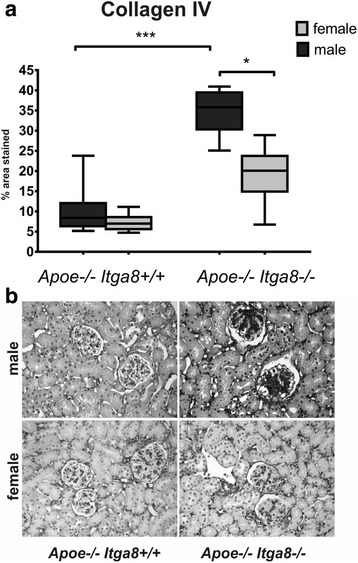
Glomerulosclerosis. **a** Evaluation of glomerulosclerosis (collagen IV staining) of male and female *Apoe* ^−/−^ *Itga8* ^+/+^ or *Apoe* ^−/−^ *Itga8* ^−/−^ mice. **b** Exemplary photomicrographs of collagen IV-stained renal sections of male and female *Apoe* ^−/−^ *Itga8* ^+/+^ or *Apoe* ^−/−^ *Itga8* ^−/−^ mice. Data are presented as means ± SEM. *p < 0.05 ***p < 0.001

**Fig. 5:**
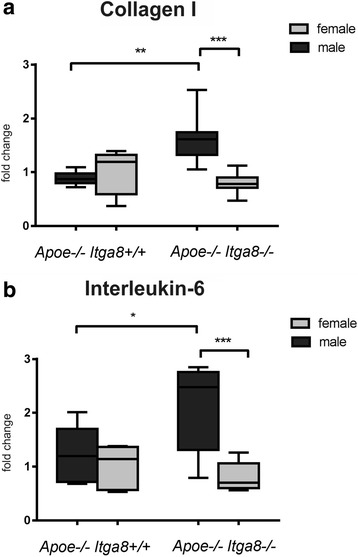
Renal markers of fibrosis and inflammation. **a** Evaluation of renal cortical collagen I expression of male and female *Apoe* ^−/−^ *Itga8* ^+/+^ or *Apoe* ^−/−^ *Itga8* ^−/−^ mice. **b** Evaluation of renal cortical interleukin-6 expression of male and female *Apoe* ^−/−^ *Itga8* ^+/+^ or *Apoe* ^−/−^ *Itga8* ^−/−^ mice. Data are presented as means ± SEM. *p < 0.05; **p < 0.01; ***p < 0.001

**Fig. 6:**
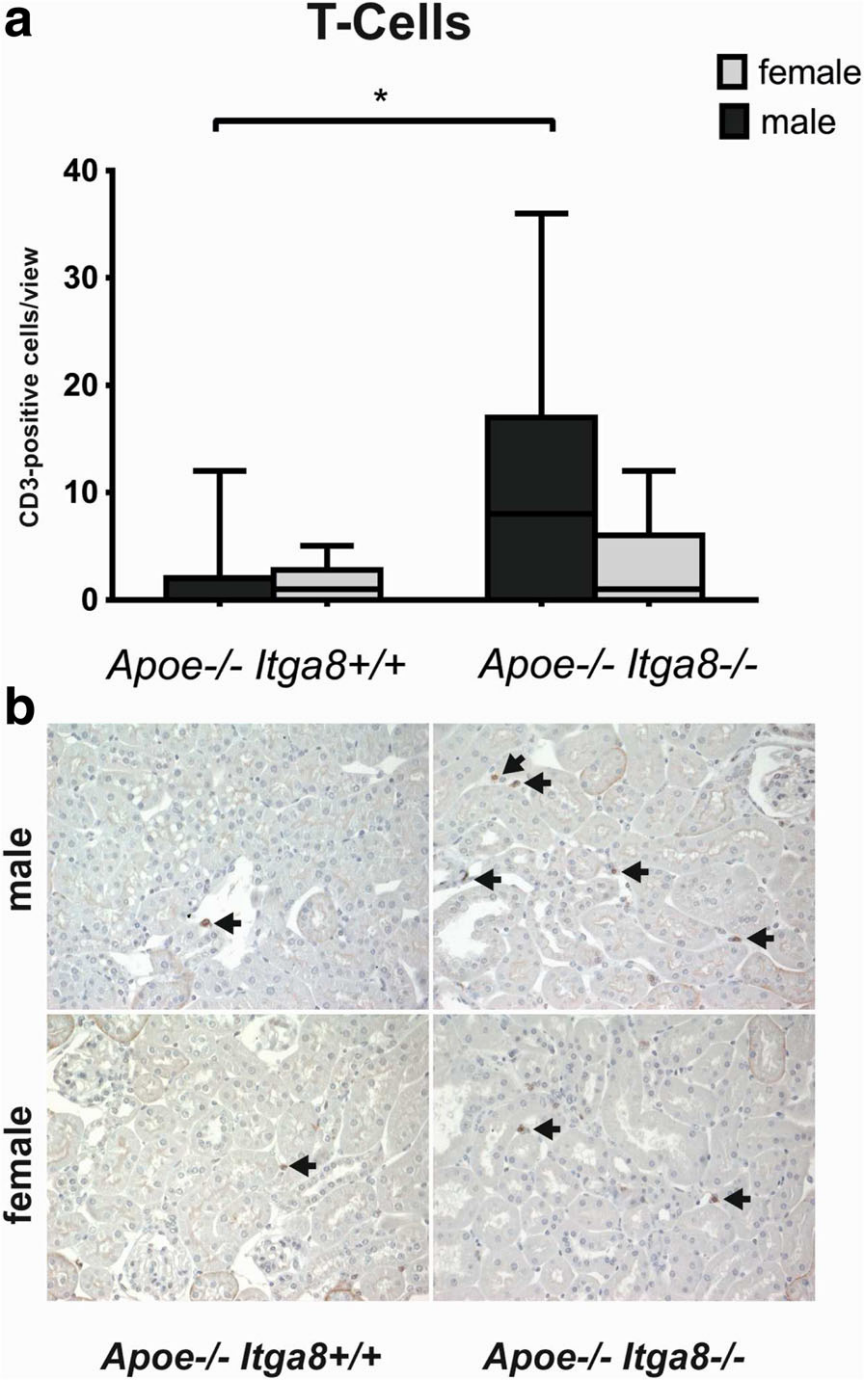
Renal T cell infiltration. **a** Evaluation of renal cortical T cell number of male and female *Apoe* ^−/−^ *Itga8* ^+/+^ or *Apoe* ^−/−^ *Itga8* ^−/−^ mice. **b** Exemplary photomicrographs of CD3-stained renal sections of male and female *Apoe* ^−/−^ *Itga8* ^+/+^ or *Apoe* ^−/−^ *Itga8* ^−/−^ mice. *Black arrows* point to CD3-positive T cells. Data are presented as means ± SEM. *p < 0.05

The deficiency of *Itga8* (*Itga8* ^−/−^) resulted in reduced renal mass and increased glomerular perimeters (Table 3) in both males and females. An increase in plasma urea as a consequence of the lack of *Apoe* and *Itga8* (*Apoe* ^−/−^ *Itga8* ^−/−^) was observed in males only (Fig. 3a). A decrease in cell turnover was likewise detected in double-knockout (*Apoe* ^−/−^ *Itga8* ^−/−^) male mice (Fig. 3b), not in females. Moreover, *Itga8* deficiency (*Itga8* ^−/−^) resulted in more pronounced glomerulosclerosis (Fig. 4) and increased cortical collagen I expression (Fig. 5a) in *Apoe*-deficient (*Apoe* ^−/−^) males as compared to females. Several renal inflammatory markers were induced in double-knockout (*Apoe* ^−/−^ *Itga8* ^−/−^) male mice. The expression of interleukin-6 (*Il6*) (Fig. 5b) and T cell infiltration (Fig. 6) was significantly higher in the kidney cortex of male compared to female mice deficient in both *Apoe* and *Itga8* (*Apoe* ^−/−^ *Itga8* ^−/−^). The expression of the chemokines CXCL-3 (*Cxcl3*, chemokine (C-X-C motif) ligand 3) and Ccl2 (*Ccl2*) tended to be higher in male mice that were deficient in *Apoe* and *Itga8* (*Apoe* ^−/−^ *Itga8* ^−/−^) when compared to females (Table 3). In contrast, renal macrophage infiltration was increased in both double-deficient (*Apoe* ^−/−^ *Itga8* ^−/−^) male and female mice to a similar degree (Table 3). Renal cortical osteopontin expression, on the other hand, was significantly higher in female mice compared to males irrespective of the genotype (Table 3). The localization of renal osteopontin in both males and females of the different genotypes was predominantly found in tubular cells (Additional file 7). Data from a two-way ANOVA of several markers of renal damage are shown in Additional file 3. A reduced level of cell turnover, as seen in male double-deficient (*Apoe* ^−/−^ *Itga8* ^−/−^) mice, might indicate increased ER stress of renal cells. Therefore, we investigated renal markers for ER stress, i.e., *Eif2ak3*, *Ddit3* (DNA damage-inducible transcript 3) and *Hspa5* (heat shock protein family A (hsp70) member 5) expression. However, none of these markers were altered in their expression in the different genotypes and sexes (Table 3). We also evaluated possible differences in renal (mRNA) *Vegfa* expression levels in male and female mice, which might account for the sex differences in renal injury. Renal (mRNA) *Vegfa* expression was somewhat higher in female *Apoe*-deficient mice compared to males of the same genotype (*Apoe* ^−/−^ *Itga8* ^+/+^). There were no sex-specific differences in (mRNA) *Vegfa* expression levels observed in male and female double-deficient (*Apoe* ^−/−^ *Itga8* ^−/−^) mice (Table 3). At 7 and 9 months of age, glomerular collagen deposition and plasma urea were already increased in male double-deficient (*Apoe* ^−/−^ *Itga8* ^−/−^) mice compared to male *Apoe* ^−/−^ *Itga8* ^+/+^. This was not observed in female mice (Additional file 6). Renal T cell infiltration was not yet different between groups at the age of 7 or 9 months (Additional file 6).

**Table 3:** Renal parameters of anatomy, inflammation, and ER stress response

	Genotype	Sex	Mean	SEM	Median	Q1	Q3	IQR
Glomerular perimeter (μm)	*Apoe* ^−/−^ *Itga8* ^+/+^	Male	202	2.47	202.5	197.5	208.9	11.4
		Female	204	3.45	204.8	196.0	213.6	17.6
	*Apoe* ^−/−^ *Itga8* ^−/−^	Male	225	5.19	225.6	209.2	241.2	32.0
		Female	234	6.51	234.0	220.0	247.3	27.3
Macrophage infiltration (no. of F4/80-pos. cells/view)	*Apoe* ^−/−^ *Itga8* ^+/+^	Male	0.92	0.52	0.0	0.0	1.0	1.0
		Female	7.17	4.21	3.5	3.5	16.5	13.0
	*Apoe* ^−/−^ *Itga8* ^−/−^	Male	53.9^#^	12.5	46.0	17.5	76.0	58.5
		Female	53.0^#^	10.7	38.0	23.0	79.0	56.0
*Spp1* mRNA expression (fold change)	*Apoe* ^−/−^ *Itga8* ^+/+^	Male	0.38	0.03	0.39	0.32	0.45	0.13
		Female	1.00*	0.07	1.04	0.87	1.12	0.25
	*Apoe* ^−/−^ *Itga8* ^−/−^	Male	0.49	0.05	0.47	0.37	0.64	0.27
		Female	1.35*	0.20	1.11	1.02	1.71	0.69
*Cxcl3* mRNA expression (fold change)	*Apoe* ^−/−^ *Itga8* ^+/+^	Male	0.75	0.18	0.66	0.39	1.22	0.83
		Female	1.00	0.29	0.91	0.42	1.63	1.21
	*Apoe* ^−/−^ *Itga8* ^−/−^	Male	2.61^#^	0.68	2.25	0.75	4.34	3.59
		Female	1.25	0.41	0.71	0.64	1.91	1.27
*Ccl2* mRNA expression (fold change)	*Apoe* ^−/−^ *Itga8* ^+/+^	Male	0.89	0.19	0.92	0.37	1.38	1.01
		Female	1.00	0.34	0.77	0.32	1.79	1.47
	*Apoe* ^−/−^ *Itga8* ^−/−^	Male	1.44	0.42	0.92	0.88	2.74	1.86
		Female	0.93	0.25	0.76	0.50	0.95	0.45
*Eif2ak3* mRNA expression (fold change)	*Apoe* ^−/−^ *Itga8* ^+/+^	Male	0.80	0.07	0.84	0.60	0.96	0.36
		Female	1.00	0.10	1.03	0.81	1.18	0.37
	*Apoe* ^−/−^ *Itga8* ^−/−^	Male	0.71	0.10	0.67	0.53	0.95	0.42
		Female	0.86	0.06	0.85	0.79	1.02	0.23
*Ddit3* mRNA expression (fold change)	*Apoe* ^−/−^ *Itga8* ^+/+^	Male	0.89	0.13	0.87	0.57	1.23	0.66
		Female	1.00	0.12	1.06	0.75	1.22	0.47
	*Apoe* ^−/−^ *Itga8* ^−/−^	Male	0.70	0.10	0.58	0.51	0.94	0.43
		Female	0.88	0.08	0.78	0.74	0.99	0.25
*Hspa5* mRNA expression (fold change)	*Apoe* ^−/−^ *Itga8* ^+/+^	Male	0.92	0.13	0.88	0.63	1.25	0.62
		Female	1.00	0.14	0.88	0.78	1.28	0.5
	*Apoe* ^−/−^ *Itga8* ^−/−^	Male	0.79	0.06	0.75	0.66	0.99	0.33
		Female	0.81	0.08	0.79	0.66	0.98	0.32
*Vegfa* mRNA expression (fold change)	*Apoe* ^−/−^ *Itga8* ^+/+^	Male	0.67	0.04	0.65	0.59	0.76	0.17
		Female	1.00	0.05	1.02	0.90	1.11	0.21
	*Apoe* ^−/−^ *Itga8* ^−/−^	Male	0.68	0.05	0.66	0.59	0.79	0.2
		Female	0.85	0.06	0.81	0.71	0.97	0.26

^#^ *p* < 0.05 vs *Itga8* ^+/+^ of the same sex

*p < 0.05 vs male of the same genotype

## Discussion

From human studies as well as from experimental data, ample evidence exists for sex-dependent differences in the development and progression of cardiovascular disease [32, 33]. The male sex is commonly recognized as an independent risk factor for cardiovascular disease [34]. In male *Apoe*-deficient mice, previous studies showed that a lack of the vascular integrin *Itga8* is associated with aggravated atherosclerosis and the development of renal injury [5]. The aim of this study was to assess sex-dependent differences in the development of vascular and renal lesions in this model of *Apoe* and *Itga8* double deficiency.

In summary, we could show that female mice that were deficient in *Apoe* (*Apoe* ^−/−^ *Itga8* ^+/+^) developed more severe atherosclerotic lesions when compared to males of the same genotype. In contrast, glomerular and interstitial lesions in the kidney were attenuated in female mice deficient in *Apoe* and *Itga8* (*Apoe* ^−/−^ *Itga8* ^−/−^) compared to male mice of the same genotype, which indicates that in this model, the female sex is protective for the development of renal injury, but not for the progression of atherosclerosis.

In our experiments, we utilized male and female *Apoe*-deficient litters on a mixed genetic background (*Apoe* ^−/−^ *Itga8* ^+/+^), which were fed regular chow, and we detected more severe vascular damage in female mice compared to males. In female *Apoe*-deficient mice, we did not observe an aggravation of atherosclerotic lesions caused by the additional underexpression of *Itga8* (*Apoe* ^−/−^ *Itga8* ^−/−^), as we saw in male *Apoe*-*Itga8* double-deficient mice [5]. These previous findings therefore seem to be limited to the male sex. Our observations of more prominent atherosclerotic alterations in female *Apoe*-deficient mice compared to males are in accordance with the findings of Caligiuri et al., which also showed more severe atherosclerotic lesions in female *Apoe*-deficient mice in comparison to males [35]. On the other hand, there are also numerous studies which report considerably more pronounced atherosclerosis in males compared to females (reviewed in [18]). The reasons for these discrepant data are presently not clear, but might involve differences in the genetic background of experimental animals. In C57BL/6 mice, for example, significantly reduced HDL levels were found under an atherogenic diet in female animals [36]. In contrast, serum levels of HDL in our *Apoe*-deficient mice on the mixed genetic background (C57BL/6x129Sv) were comparable between the sexes. Similar divergent observations were made with triglyceride levels in *Apoe*-deficient mice on the C57BL/6 or Ola129 genetic backgrounds [21]. Furthermore, in our study, *Apoe*-deficient animals were not exposed to an atherogenic diet, which seems to be a relevant stimulus for HDL regulation in these mice [36]. Taken together, sex-dependent regulation of serum fat levels does not seem to be the underlying mechanism leading to more severe atherosclerosis in female *Apoe*-deficient mice in our study. Sex hormones may exert protective roles in the development of atherosclerosis. While it is widely accepted that estrogens have protective effects on the cardiovascular system [37], testosterone levels in the physiological range are also assumed to have protective effects [38]. In this context, the age-dependent decrease in testosterone levels and associated metabolic alterations are relevant pathogenetic factors for the development of atherosclerosis in men [39, 40]. Vasculoprotective effects of testosterone were also shown in animal studies. Thus, the less severe atherosclerotic changes in male *Apoe*-deficient mice compared to females might be caused by beneficial effects of testosterone.

Hyperlipidemia is associated with an elevated risk for the development of kidney disease [41, 42]. Numerous clinical and animal studies suggest that *Apoe* deficiency can be accompanied by renal disease [4, 43]. In our model of *Apoe* deficiency, however, overt renal injury did not develop without an additional challenge, like the concomitant deficiency of *Itga8* [5]. In male mice with an *Apoe*-*Itga8* double deficiency (*Apoe* ^−/−^ *Itga8* ^−/−^), we detected marked glomerulosclerosis [5], which was clearly attenuated by the female sex, as shown in the present study. Different hormone levels in male and female mice might affect the susceptibility to develop renal injury, as some evidence exists that testosterone action in the kidney may contribute to glomerulosclerosis [44, 45].

ER stress response was shown to play a crucial role in the development of atherosclerosis and kidney disease [46, 47]. The chaperone CHOP (synonym *Ddit3*) was found as a target to modulate the severity of atherosclerotic lesions in *Apoe*-deficient mice [48]. Moreover, sex differences in the induction of ER stress biomarker expression were detected in experimental hypertension [49]. Therefore, we tested the hypothesis that ER stress parameters might be differentially regulated in our male and female *Apoe*-*Itga8* double-deficient (*Apoe* ^−/−^ *Itga8* ^−/−^) mice. In our study, the expression patterns of the chaperones *Ddit3*, *Eif2ak3*, and *Hspa5* were comparable in males and females, arguing against a relevant role for ER stress in sex differences during the development of kidney damage in *Apoe*-*Itga8* double-deficient (*Apoe* ^−/−^ *Itga8* ^−/−^) mice.

In a rat study of chronic renal disease, female rats were protected from the progression of renal injury by estrogen-stimulated *Vegfa* expression [50]. In contrast, VEGF seemed to be detrimental for atherosclerotic plaque progression [51]. We therefore tested *Vegfa* expression in our model. *Vegfa* expression was somewhat higher in female *Apoe*-deficient (*Apoe* ^−/−^ *Itga8* ^+/+^) mice compared to males. However, *Vegfa* expression levels of *Apoe*-*Itga8* double-deficient (*Apoe* ^−/−^ *Itga8* ^−/−^) were not different between males and females and can therefore not explain the higher susceptibility for renal injury in male mice of this genotype.

Several studies detected sex differences in the inflammatory response during cardiovascular disease [52, 53]. Ji et al. showed a sex-dependent regulation of T cells and T cell-mediated functions in an animal model of hypertension with an induced activation of T cells in males [54]. T cells seem to have important functions in the development of atherosclerosis of *Apoe*-deficient mice. Laskowitz et al. showed an impaired T cell-associated immune response [55]. Robert et al. described a higher renal T cell infiltration rate in male animals after experimental kidney ischemia-reperfusion injury compared to females [56]. In our study, a significantly higher T cell infiltration of the kidney from male *Apoe*-*Itga8* double-deficient (*Apoe* ^−/−^ *Itga8* ^−/−^) animals compared to females was also detected. This notion is supported by concordantly altered expression patterns of the cytokines *Il6* and *Cxcl3*, arguing for differences in the inflammatory response in male and female *Apoe*-*Itga8* double-deficient (*Apoe* ^−/−^ *Itga8* ^−/−^) mice. These differences might contribute to the more severe renal injury in male mice compared to females.

In our experimental model, we observed higher osteopontin levels in female mice of both genotypes (*Apoe* ^−/−^ *Itga8* ^+/+^ and *Apoe* ^−/−^ *Itga8* ^−/−^) compared to males. Osteopontin (*Spp1*) is known to be regulated by sex hormones: Estrogen can induce osteopontin expression [57], while testosterone is able to suppress it [58]. Osteopontin is a multifunctional protein contributing to the regulation of inflammation, atherosclerosis, and vascular calcification [59]. In accordance with our observations, osteopontin was described to be present in calcified vessels [60]. Whether osteopontin is beneficial or detrimental in atherosclerosis is controversial, but it seems that osteopontin contributes to the onset of aortic calcification, while it inhibits the progression of atherosclerosis [61]. Matsui et al. found that osteopontin deficiency attenuated atherosclerosis in female *Apoe*-deficient mice [62]. We therefore speculate that high levels of vascular osteopontin might contribute to the more severe atherosclerotic lesions detected in our female *Apoe*-deficient mice (*Apoe* ^−/−^ *Itga8* ^+/+^ and *Apoe* ^−/−^ *Itga8* ^−/−^). Although the action of osteopontin in the kidney is still somewhat controversial [63], several studies found renoprotective effects of osteopontin [64–66]. This might explain the protection from renal damage observed in our female *Apoe*-*Itga8* double-deficient (*Apoe* ^−/−^ *Itga8* ^−/−^) mice.

## Conclusions

Taken together, we observed that in the *Apoe*-deficient model of atherosclerosis, the female sex is a risk factor to develop more severe atherosclerotic lesions, although serum fat levels are not higher in females than in males. In contrast, female mice are protected from renal damage induced by the concomitant deficiency of *Apoe* and *Itga8*. These differences might be due to sex differences in the inflammatory response and/or osteopontin levels. Thus, sex affects vascular and renal injury not uniformly but in a differential manner. The reason for these differential outcomes is not clear by now, but our results suggest that there is more than one risk factor contributing to the development of vascular or renal lesions in our animal model of hyperlipidemia. The development of organ damage seems to be a result of an interplay of sex, genotype, serum lipids, and inflammatory mediators, which might affect organs differentially.

### Limitation

In our study, we used mice on a mixed genetic background. Therefore, our data cannot readily be extrapolated to data that has been collected in similar studies performed with mice on a pure genetic background.

## Additional files

Additional file 1: Primer pairs used in the study. (DOCX 24 kb)

Additional file 2: Anatomical and metabolic parameters of mice without a deletion in *Apoe* (*Apoe* ^+/+^ *Itga8* ^+/+^ or *Apoe* ^++^ *Itga8* ^−/−^). (DOCX 22 kb)

Additional file 3: Results from two-way ANOVA of markers of vascular and renal injury. (DOCX 19 kb)

Additional file 4: Atherosclerotic plaque formation in mice without a deficiency of *Apoe*. Exemplary en face preparations of aortae of male and female *Apoe* ^+/+^ *Itga8* ^+/+^ or *Apoe* ^+/+^ *Itga8* ^−/−^ mice, stained with Sudan IV (left) or unstained (right). One en face preparation of the aorta of an *Apoe*-deficient (*Apoe* ^−/−^ *Itga8* ^+/+^) female mouse was stained for Sudan IV as a positive control. (PDF 257 kb)

Additional file 5: Osteopontin in the brachiocephalic trunk. Exemplary osteopontin-stained cross sections of the brachiocephalic trunk of male and female mice with a deficiency of *Apoe*. (PDF 303 kb)

Additional file 6: Vascular and renal changes in 7- and 9-month-old mice. (DOCX 25 kb)

Additional file 7: Osteopontin in the kidney. Exemplary photomicrographs of osteopontin-stained renal sections of male and female *Apoe* ^−/−^ *Itga8* ^+/+^ or *Apoe* ^−/−^ *Itga8* ^−/−^ mice. (PDF 548 kb)

## Abbreviations

*Actb*: Beta actin, gene
*Apoe*: Apolipoprotein E, gene
*Apoe*^−/−^: *Apoe*-deficient
*Ccl2*: CC-chemokine ligand 2, gene
*Col1a1*: Collagen 1, gene
*Cxcl3*: Chemokine (C-X-C motif) ligand 3, gene
*Ddit3*: DNA-damage inducible transcript 3, gene
*Eif2ak3*: Eukaryotic translation initiation factor 2-alpha kinase 3, gene
*Gapdh*: Glyceraldehyde 3-phosphate dehydrogenase, gene
HDL: High-density lipoprotein
*Hspa5*: Heat shock protein family A (hsp70) member 5, gene
*Il6*: Interleukin-6, gene
*Itga8*: Integrin alpha 8, gene
*Itga8*^−/−^: *Itga8*-deficient
LC–MS/MS: Liquid chromatography-tandem mass spectrometry
LDL: Low-density lipoprotein
PCNA: Proliferating cell nuclear antigen
*Rn18s*: 18S rRNA, gene
SEM: Standard error of the mean
SPE-HPLC-MS/MS: Solid-phase extraction-liquid chromatography-tandem mass spectrometry
*Spp1*: Secreted phosphoprotein 1, gene
*Vegfa*: Vascular endothelial growth factor, gene
