# Supplementary material for: Sex differences in the development of vascular and renal lesions in mice with a simultaneous deficiency of Apoe and the integrin chain Itga8
Source: Biol Sex Differ. 2017 May 30;8:19. doi: 10.1186/s13293-017-0141-y (PMC5450388; doi:10.1186/s13293-017-0141-y)
Supplement: Supplementary file 1 — Primer pairs used in the study. (DOCX 24 kb) [file 13293_2017_141_MOESM1_ESM.docx]

**Additional file 1: Primer pairs used for Real-time PCR analysis**

| **gene** | **sequence** | **accession number** | **amplicon length** |
| --- | --- | --- | --- |
| ***Col1a1*** | fw 5‘-TCACCTACAGCACCCTTGTGG-3‘  rv 5‘-CCCAAGTTCCGGTGTGACTC-3‘ | NM_007742.4 | 51 |
| ***Ddit3*** | fw 5‘-GAAGAGGAAGAATCAAAAACCTTCA-3‘  rv 5‘-ATGTGCGTGTGACCTCTGTTG-3‘ | NM_007837.4 | 94 |
| ***Cxcl3*** | fw 5‘-TTTGAGACCATCCAGAGCTTGA-3‘  rv 5‘-CCTTGAGAGTGGCTATGACTTCTGT-3’ | NM_203320.3 | 79 |
| ***Hspa5*** | fw 5‘-GCCTCATCGGACGCACTT-3‘  rv 5‘-AACCACCTTGAATGGCAAGAA-3‘ | NM_001163434.1 | 71 |
| ***Il6*** | fw 5‘-CCACGGCCTTCCCTACTTC-3‘  rv 5‘-TGCACAACTCTTTTCTCATTTCCA-3‘ | NM_031168.1 | 147 |
| ***Itga8*** | fw 5‘-AGAATGATTACCCAGATTTGCTTGT-3‘  rv 5‘-GCTACTTTCCCTTTTCCAAATGC-3‘ | NM_001001309.2 | 52 |
| ***Spp1*** | fw 5‘-TTTGCTTTTGCCTGTTTGGC-3‘  rv 5‘-CAGTCACTTTCACCGGGAGG-3‘ | NM_001204201.1 | 51 |
| ***Ccl2*** | fw 5‘-CCACTCACCTGCTGCTACTCAT-3‘  rv 5‘-TGGTGATCCTCTTGTAGCTCTCC-3‘ | NM_011333.3 | 76 |
| ***Eif2ak3*** | fw 5‘-CCCACAGGCAGCGGAAG-3‘  rv 5‘-GTCACTGACATCGGCACTCA-3‘ | NM_010121.3 | 80 |
| ***Vegfa*** | fw 5‘-AACGATGAAGCCCTGGAGTG-3‘  rv 5‘-TGAGAGGTCTGGTTCCCGA-3‘ | NM_001287056.1 variant 1 | 440 |
| ***Rn18s*** | fw 5‘-TTGATTAAGTCCCTGCCCTTTGT-3‘  rv 5‘-CGATCCGAGGGCCTCACTA-3‘ | XR_877120.2 | 78 |
| **Actb** | fw 5‘-GGCGCTTTTGACTCAGGATT-3‘  rv 5‘-GGGATGTTTGCTCCAACCAA-3‘ | NM007393.5 | 70 |
| **Gapdh** | fw 5‘-TGTGTCCGTCGTGGATCTGA-3‘  rv 5‘-CCTGCTTCACCACCTTCTTGA-3‘ | NM001289726.1 | 77 |
