# Supplementary material for: Sex differences in the development of vascular and renal lesions in mice with a simultaneous deficiency of Apoe and the integrin chain Itga8
Source: Biol Sex Differ. 2017 May 30;8:19. doi: 10.1186/s13293-017-0141-y (PMC5450388; doi:10.1186/s13293-017-0141-y)
Supplement: Supplementary file 2 — Anatomical and metabolic parameters of mice without a deletion in Apoe (Apoe +/+ Itga8 +/+ or Apoe ++ Itga8 −/−). (DOCX 22 kb) [file 13293_2017_141_MOESM2_ESM.docx]

**Additional file 2: Anatomical and metabolic parameters of control mice without a deletion in *Apoe***

|  | **Genotype** | **Sex** | **Mean** | **SEM** | **Median** | **Q1** | **Q3** | **IQR** |
| --- | --- | --- | --- | --- | --- | --- | --- | --- |
| **Body weight [g]** | ***Apoe*^+/+^*Itga8*^+/+^** | male | 31.2 | 0.7 | 31.0 | 29.8 | 32.7 | 2.9 |
|  |  | female | 24.7* | 0.6 | 24.8 | 22.6 | 26. 8 | 4.2 |
|  | ***Apoe*^+/+^*Itga8*^-/-^** | male | 29.5 | 0.7 | 28.8 | 28.2 | 31.0 | 2.8 |
|  |  | female | 23.9* | 1.0 | 23.6 | 22.1 | 25.5 | 3.4 |
| **Absolute kidney weight [g]** | ***Apoe*^+/+^*Itga8*^+/+^** | male | 0.38 | 0.02 | 0.38 | 0.33 | 0.42 | 0.09 |
|  |  | female | 0.27* | 0.01 | 0.26 | 0.25 | 0.29 | 0.04 |
|  | ***Apoe*^+/+^*Itga8*^-/-^** | male | 0.27^#^ | 0.02 | 0.26 | 0.22 | 0.28 | 0.06 |
|  |  | female | 0.21 | 0.02 | 0.20 | 0.17 | 0.24 | 0.07 |
| **Relative kidney weight [%]** | ***Apoe*^+/+^*Itga8*^+/+^** | male | 1.24 | 0.05 | 1.19 | 1.14 | 1.38 | 0.24 |
|  |  | female | 1.07* | 0.04 | 1.07 | 0.94 | 1.21 | 0.27 |
|  | ***Apoe*^+/+^*Itga8*^-/-^** | male | 0.92^#^ | 0.06 | 0.91 | 0.77 | 0.96 | 0.19 |
|  |  | female | 0.85^#^ | 0.05 | 0.88 | 0.77 | 0.97 | 0.2 |
| **Relative left ventricular weight [%]** | ***Apoe*^+/+^*Itga8*^+/+^** | male | 0.43 | 0.03 | 0.40 | 0.39 | 0.48 | 0.09 |
|  |  | female | 0.39 | 0.02 | 0.39 | 0.34 | 0.43 | 0.09 |
|  | ***Apoe*^+/+^*Itga8*^-/-^** | male | 0.38 | 0.02 | 0.38 | 0.36 | 0.43 | 0.07 |
|  |  | female | 0.40 | 0.02 | 0.39 | 0.37 | 0.41 | 0.08 |
| **Plasma urea [mg/dl]** | ***Apoe*^+/+^*Itga8*^+/+^** | male | 62 | 4 | 59 | 51 | 71 | 19 |
|  |  | female | 59 | 7 | 59 | 44 | 65 | 21 |
|  | ***Apoe*^+/+^*Itga8*^-/-^** | male | 84 | 5 | 83 | 75 | 94 | 19 |
|  |  | female | 123 | 49 | 76 | 63 | 92 | 30 |
| **Plasma triglycerides [mg/dl]** | ***Apoe*^+/+^*Itga8*^+/+^** | male | 77 | 8 | 79 | 65 | 99 | 34 |
|  |  | female | 57 | 9 | 44 | 37 | 76 | 39 |
|  | ***Apoe*^+/+^*Itga8*^-/-^** | male | 88 | 12 | 92 | 47 | 122 | 75 |
|  |  | female | 83 | 15 | 60 | 48 | 146 | 98 |
| **Plasma total cholesterol [mg/dl]** | ***Apoe*^+/+^*Itga8*^+/+^** | male | 84 | 7 | 81 | 69 | 110 | 41 |
|  |  | female | 76 | 8 | 66 | 58 | 91 | 33 |
|  | ***Apoe*^+/+^*Itga8*^-/-^** | male | 97 | 7 | 103 | 64 | 114 | 49 |
|  |  | female | 84 | 4 | 84 | 73 | 93 | 20 |
| **Plasma HDL cholesterol [mg/dl]** | ***Apoe*^+/+^*Itga8*^+/+^** | male | 72 | 6 | 71 | 55 | 96 | 41 |
|  |  | female | 66 | 8 | 60 | 53 | 68 | 15 |
|  | ***Apoe*^+/+^*Itga8*^-/-^** | male | 84 | 7 | 89 | 56 | 101 | 45 |
|  |  | female | 71 | 3 | 69 | 64 | 79 | 15 |

# p<0.05 vs *Itga8*^+/+^ of same sex

* p<0.05 vs male of same genotype
