# Supplementary material for: Sex differences in the development of vascular and renal lesions in mice with a simultaneous deficiency of Apoe and the integrin chain Itga8
Source: Biol Sex Differ. 2017 May 30;8:19. doi: 10.1186/s13293-017-0141-y (PMC5450388; doi:10.1186/s13293-017-0141-y)
Supplement: Supplementary file 3 — Results from two-way ANOVA of markers of vascular and renal injury. (DOCX 19 kb) [file 13293_2017_141_MOESM3_ESM.docx]

| **Random factor** | **Fixed factors** | **F-value** | **df1, df2** | **p-value** | **η^2^** |
| --- | --- | --- | --- | --- | --- |
| **Aortic lipid deposition** | sex | 0.746 | 1,48 | 0.392 |  |
|  | genotype | 1.221 | 1,48 | 0.275 |  |
|  | sex*genotype | 9.305 | 1,48 | **0.004** | 0.162 |
| **Aortic calcification** | sex | 17.297 | 1,48 | **< 0.001** | 0.265 |
|  | genotype | 0.364 | 1,48 | 0.549 |  |
|  | sex*genotype | 0.223 | 1,48 | 0.639 |  |
| ***Cxcl3* (mRNA) expression** | sex | 1.367 | 1,21 | 0.255 |  |
|  | genotype | 4.926 | 1,21 | **0.038** | 0.190 |
|  | sex*genotype | 2.849 | 1,21 | 0.106 |  |
| ***Spp1* (mRNA) expression** | sex | 79.579 | 1,19 | **< 0.001** | 0.807 |
|  | genotype | 3.647 | 1,19 | 0.071 |  |
|  | sex*genotype | 0.159 | 1,19 | 0.695 |  |
| ***Col1a1*  (mRNA) expression** | sex | 6.887 | 1,21 | **0.016** | 0.247 |
|  | genotype | 3.695 | 1,21 | 0.068 |  |
|  | sex*genotype | 12.173 | 1,21 | **0.002** | 0.367 |
| ***Il6* (mRNA) expression** | sex | 12.339 | 1,21 | **0.002** | 0.37 |
|  | genotype | 2.347 | 1,21 | 0.14 |  |
|  | sex*genotype | 5.950 | 1,21 | **0.024** | 0.221 |
| **Collagen IV staining** | sex | 30.123 | 1,41 | **< 0.001** | 0.424 |
|  | genotype | 128.942 | 1,41 | **< 0.001** | 0.759 |
|  | sex*genotype | 14.551 | 1,41 | **< 0.001** | 0.262 |
| **PCNA staining** | sex | 0.581 | 1,35 | 0.451 |  |
|  | genotype | 7.063 | 1,35 | **0.012** | 0.168 |
|  | sex*genotype | 6.242 | 1,35 | **0.017** | 0.151 |
| **CD3 staining** | sex | 3.085 | 1,42 | 0.086 |  |
|  | genotype | 5.695 | 1,42 | **0.022** | 0.119 |
|  | sex*genotype | 2.949 | 1,42 | 0.093 |  |
| **Plasma Urea** | sex | 1.619 | 1,72 | 0.207 |  |
|  | genotype | 24.155 | 1,72 | **< 0.001** | 0.251 |
|  | sex*genotype | 5.045 | 1,72 | **0.028** | 0.065 |

**Additional file 3: Results from two way ANOVA of markers of vascular and renal injury.**
