# Supplementary material for: Sex differences in the development of vascular and renal lesions in mice with a simultaneous deficiency of Apoe and the integrin chain Itga8
Source: Biol Sex Differ. 2017 May 30;8:19. doi: 10.1186/s13293-017-0141-y (PMC5450388; doi:10.1186/s13293-017-0141-y)
Supplement: Supplementary file 4 — Atherosclerotic plaque formation in mice without a deficiency of Apoe. Exemplary en face preparations of aortae of male and female Apoe +/+ Itga8 +/+ or Apoe +/+ Itga8 −/− mice, stained with Sudan IV (left) or unstained (right). One en face preparation of the aorta of an Apoe-deficient (Apoe −/− Itga8 +/+) female mouse was stained for Sudan IV as a positive control. (PDF 257 kb) [file 13293_2017_141_MOESM4_ESM.pdf]

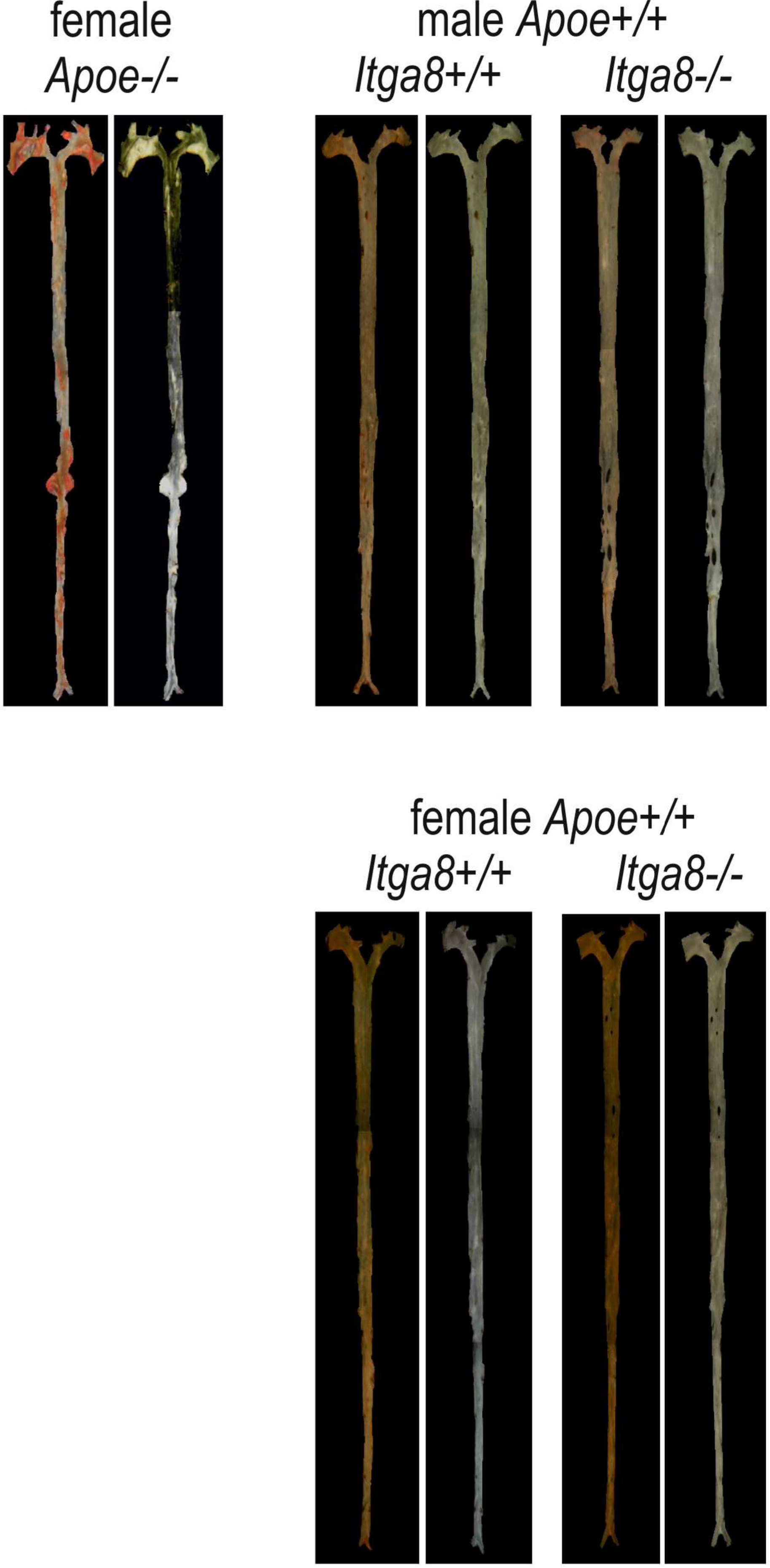

Additional file 4: Atherosclerotic plaque formation in mice without a deficiency for *Apoe*. Exemplary en face preparations of aortae of male and female *Apoe+/+ Itga8+/+* or *Apoe+/+ Itga8-/-* mice, stained with sudan IV (left) or unstained (right). One en face preparation of the aorta of an *Apoe*-deficient female mouse was stained for sudan IV as a positive control.
