# Supplementary material for: Sex differences in the development of vascular and renal lesions in mice with a simultaneous deficiency of Apoe and the integrin chain Itga8
Source: Biol Sex Differ. 2017 May 30;8:19. doi: 10.1186/s13293-017-0141-y (PMC5450388; doi:10.1186/s13293-017-0141-y)
Supplement: Supplementary file 5 — Osteopontin in the brachiocephalic trunk. Exemplary osteopontin-stained cross sections of the brachiocephalic trunk of male and female mice with a deficiency of Apoe. (PDF 303 kb) [file 13293_2017_141_MOESM5_ESM.pdf]

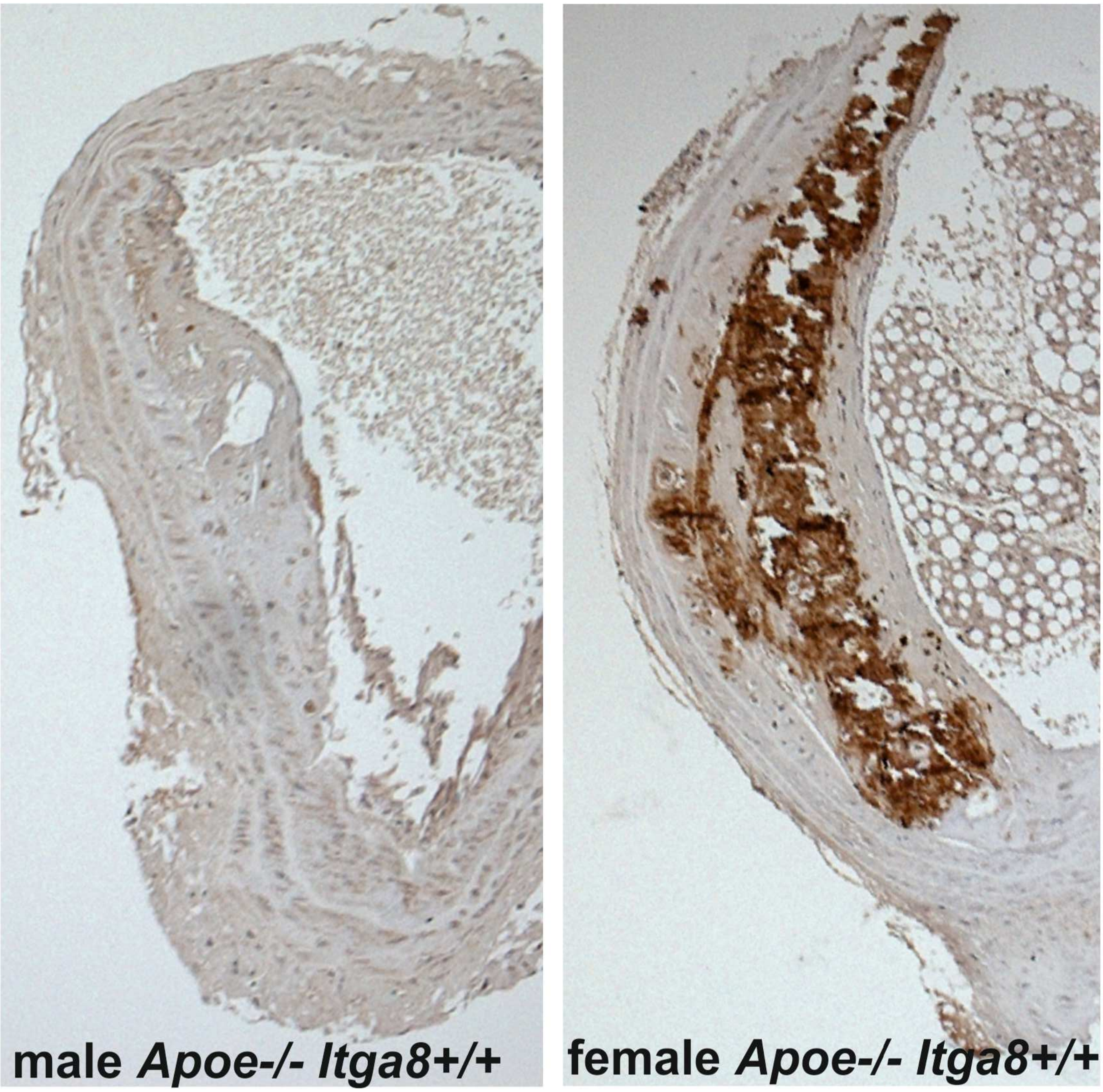

Additional file 5: Osteopontin in the brachiocephalic trunk.
Exemplary osteopontin-stained cross sections of the brachiocephalic trunk of male and female mice with a deficiency for *Apoe*
