# Supplementary material for: Sex differences in the development of vascular and renal lesions in mice with a simultaneous deficiency of Apoe and the integrin chain Itga8
Source: Biol Sex Differ. 2017 May 30;8:19. doi: 10.1186/s13293-017-0141-y (PMC5450388; doi:10.1186/s13293-017-0141-y)
Supplement: Supplementary file 6 — Vascular and renal changes in 7- and 9-month-old mice. (DOCX 25 kb) [file 13293_2017_141_MOESM6_ESM.docx]

| **7 months** | **Genotype** | **Sex** | **Mean** | **SEM** | **Median** | **Q1** | **Q3** | **IQR** |
| --- | --- | --- | --- | --- | --- | --- | --- | --- |
| **Plaques in aorta**  **[% area]** | ***Apoe*^-/-^ *Itga8*^+/+^** | male | 1.18 | 0.32 | 1.48 | 0.23 | 1.64 | 1.41 |
|  |  | female | 2.71 | 0.58 | 2.55 | 1.43 | 4.23 | 2.80 |
|  | ***Apoe*^-/-^ *Itga8*^-/-^** | male | 2.23 | 0.70 | 1.52 | 0.97 | 4.29 | 3.32 |
|  |  | female | 1.89 | 0.51 | 1.59 | 1.00 | 3.08 | 2.08 |
| **Glomerular collagen IV [% area]** | ***Apoe*^-/-^ *Itga8*^+/+^** | male | 6.02 | 2.39 | 3.90 | 2.85 | 10.25 | 7.4 |
|  |  | female | 7.61 | 0.62 | 7.44 | 6.70 | 9.12 | 2.42 |
|  | ***Apoe*^-/-^ *Itga8*^-/-^** | male | 20.68^#^ | 2.23 | 20.8 | 15.9 | 25.4 | 9.5 |
|  |  | female | 10.96 | 0.56 | 11.16 | 9.69 | 12.12 | 2.43 |
| **Plasma urea [mg/dl]** | ***Apoe*^-/-^ *Itga8*^+/+^** | male | 53 | 2 | 50 | 47 | 59 | 12 |
|  |  | female | 49 | 3 | 50 | 37 | 59 | 22 |
|  | ***Apoe*^-/-^ *Itga8*^-/-^** | male | 86^#^ | 10 | 84 | 67 | 115 | 48 |
|  |  | female | 57 | 3 | 57 | 46 | 65 | 19 |
| **Renal T-cell infiltration [no/view]** | ***Apoe*^-/-^ *Itga8*^+/+^** | male | 0.68 | 0.10 | 0.75 | 0.45 | 0.85 | 0.4 |
|  |  | female | 0.52 | 0.29 | 0.2 | 0.08 | 1 | 0.92 |
|  | ***Apoe*^-/-^ *Itga8*^-/-^** | male | 1.08 | 0.33 | 1 | 0.28 | 1.95 | 1.68 |
|  |  | female | 1.13 | 0.26 | 0.95 | 0.68 | 1.55 | 0.88 |

**Additional file 6: Vascular and renal changes of 7 and 9 months old mice.**

| **9 months** | **Genotype** | **Sex** | **Mean** | **SEM** | **Median** | **Q1** | **Q3** | **IQR** |
| --- | --- | --- | --- | --- | --- | --- | --- | --- |
| **Plaques in aorta**  **[% area]** | ***Apoe*^-/-^ *Itga8*^+/+^** | male | 1.65 | 0.53 | 0.96 | 0.71 | 3.11 | 2.40 |
|  |  | female | 5.5* | 0.84 | 5.44 | 3.85 | 7.26 | 3.41 |
|  | ***Apoe*^-/-^ *Itga8*^-/-^** | male | 5.53^#^ | 0.98 | 4.22 | 3.27 | 8.23 | 4.96 |
|  |  | female | 4.45 | 0.68 | 4.03 | 2.93 | 6.40 | 3.47 |
| **Glomerular collagen IV [% area]** | ***Apoe*^-/-^ *Itga8*^+/+^** | male | 8.06 | 0.89 | 7.71 | 6.19 | 10.1 | 3.91 |
|  |  | female | 9.24 | 0.59 | 9.28 | 7.96 | 10.74 | 2.78 |
|  | ***Apoe*^-/-^ *Itga8*^-/-^** | male | 21.3^#^ | 3.78 | 10.76 | 14.36 | 28.87 | 14.51 |
|  |  | female | 10.96* | 0.56 | 11.16 | 9.69 | 12.12 | 2.43 |
| **Plasma urea [mg/dl]** | ***Apoe*^-/-^ *Itga8*^+/+^** | male | 50 | 2 | 49 | 44 | 54 | 10 |
|  |  | female | 51 | 3 | 54 | 47 | 57 | 9 |
|  | ***Apoe*^-/-^ *Itga8*^-/-^** | male | 72 | 5 | 69 | 59 | 84 | 25 |
|  |  | female | 57 | 5 | 59 | 45 | 68 | 23 |
| **Renal T-cell infiltration [no/view]** | ***Apoe*^-/-^ *Itga8*^+/+^** | male | 1.10 | 0.27 | 1.40 | 0.45 | 1.60 | 1.15 |
|  |  | female | 1.37 | 0.38 | 1.40 | 0.53 | 2.23 | 1.70 |
|  | ***Apoe*^-/-^ *Itga8*^-/-^** | male | 1.50 | 0.49 | 1.30 | 0.68 | 2.53 | 1.85 |
|  |  | female | 0.85 | 0.27 | 0.90 | 0.10 | 1.53 | 1.43 |

# p<0.05 vs *Itga8*^+/+^ of same sex

* p<0.05 vs male of same genotype
