# Supplementary material for: Sex differences in the development of vascular and renal lesions in mice with a simultaneous deficiency of Apoe and the integrin chain Itga8
Source: Biol Sex Differ. 2017 May 30;8:19. doi: 10.1186/s13293-017-0141-y (PMC5450388; doi:10.1186/s13293-017-0141-y)
Supplement: Supplementary file 7 — Osteopontin in the kidney. Exemplary photomicrographs of osteopontin-stained renal sections of male and female Apoe −/− Itga8 +/+ or Apoe −/− Itga8 −/− mice. (PDF 548 kb) [file 13293_2017_141_MOESM7_ESM.pdf]

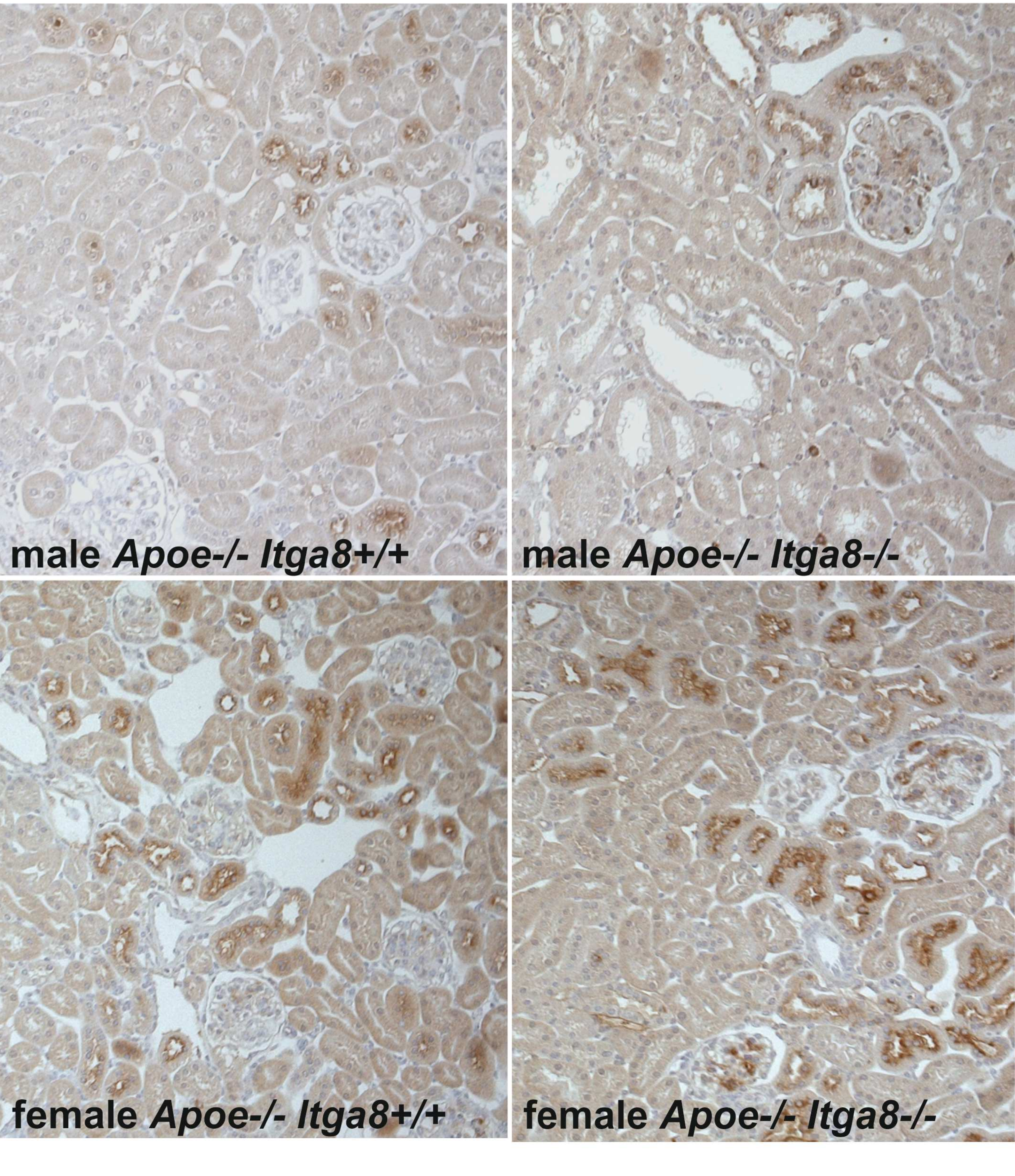

Additional file 7: Osteopontin in the kidney.
Exemplary photomicrographs of osteopontin-stained renal sections of male and female *Apoe-/- Itga8+/+* or *Apoe-/- Itga8-/-* mice.
